# A case of left ventricular pseudoaneurysm presenting with a visible apex beat

**DOI:** 10.1093/ehjcr/yty052

**Published:** 2018-04-24

**Authors:** Shinichiro Masuda, Takashi Shibui, Ryoko Onodera, Takashi Ashikaga

**Affiliations:** 1 Department of Cardiology, Tokyo Metropolitan Hiroo Hospital, 2-34-10, Ebisu, Shibuya-ku, Tokyo, Japan; 2 Department of Cardiology, Tokyo Medical and Dental University Hospital, 1-5-4 Yushima, Bunkyo-ku, Tokyo, Japan

## Case description

**Figure 1:**
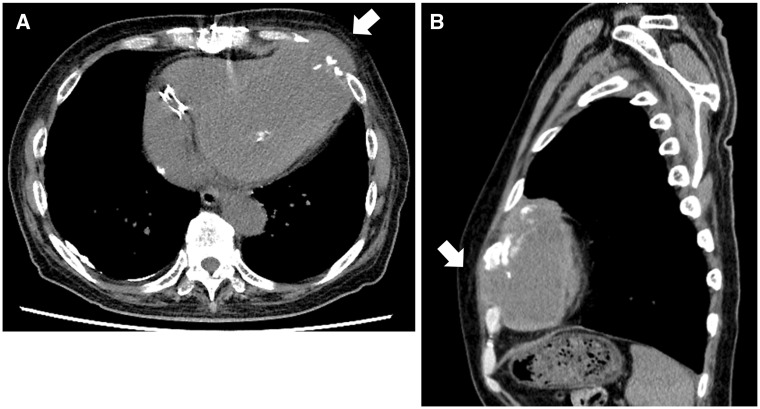
Computed tomography shows a pseudoaneurysm (white arrow) at the apex of the heart, which protruding outside of the thorax. (*A*) Transverse view. (*B*) Sagittal view.

**Figure 2:**
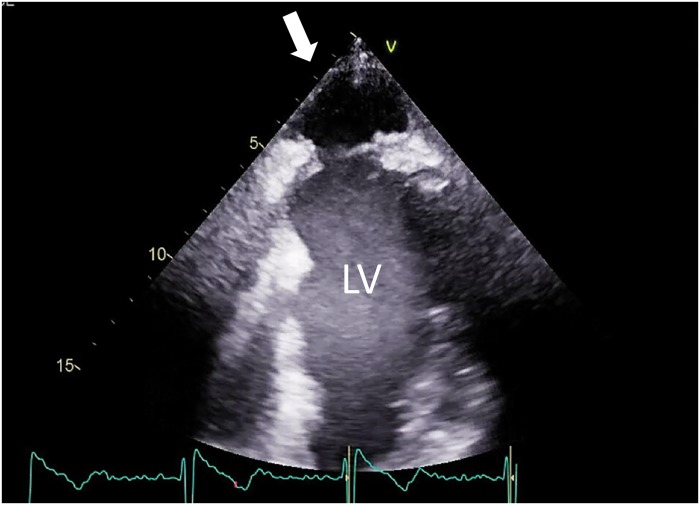
Transthoracic echocardiogram in a two-chamber view shows a 40 × 27-mm pseudoaneurysm (white arrow), connecting with the left ventricle around the apex of the heart. LV, left ventricle.

A 72-year-old man with a history of anteroseptal acute myocardial infarction was admitted to our hospital with acute heart failure. He underwent left ventricular (LV) reconstruction due to LV aneurysm, coronary artery bypass grafting from the left internal thoracic artery to the left anterior descending artery, mitral valvuloplasty with artificial ring for moderate to severe mitral regurgitation, and tricuspid valvuloplasty with artificial ring 18 months earlier. On admission, his blood pressure was 86/55 mmHg, heart rate was 60 b.p.m., and a visible apex beat was present at the 6th left intercostal space just medial to the left mid-clavicular line (Supplementary material online, *Video S1*). Computed tomography revealed a pseudoaneurysm with mild calcification, protruding outside the thorax around the apex of the heart (*Figure 1*). Echocardiogram showed akinesis of the anteroseptal wall and a 40 × 27-mm pseudoaneurysm around the apex of the heart, moving in synchrony with the heartbeat, and a 17% LV ejection fraction (*Figure 2*, Supplementary material online, *Video S2*). The connection was confirmed between the pseudoaneurysm and the left ventricle with a spontaneous echo contrast. An underlying infection could have contributed to the formation of the pseudoaneurysm. We theorize that as the pseudoaneurysm progressed, the tissue along its path of progression was mechanically damaged. This gradual damage could have resulted in impaired structural integrity of the surrounding tissue which ultimately allowed certain segments of the heart to protrude into regions beyond the normal anatomical confines of the heart. Left ventricular ejection fraction slightly improved from 17% to 25%, before and after the operation, respectively. The patient was discharged after a protracted hospital course. After discharge, the patient is now coming for follow-ups at regular intervals. Most LV pseudoaneurysm patients present with various clinical observations and abnormal findings on physical examination, such as heart failure, dyspnoea, and chest pain; however, 10% of patients are asymptomatic.^1^ In the present case, the pseudoaneurysm protruded into the left thoracic cavity with the pericardium and perforated subcutaneously from the intercostal space. Post-myocardial infarction LV pseudoaneurysm has been reported^2^^,^^3^; however, a pseudoaneurysm presenting with a visible apex beat is exceptionally rare.

## Supplementary material

Supplementary material is available at *European Heart Journal - Case Reports* online.

**Consent:** The author/s confirm that written consent for submission and publication of this case report including image(s) and associated text has been obtained from the patient in line with COPE guidance.

**Conflict of interest:** none declared.

## Supplementary Material

Supplementary Data
